# Intersection Between Local Anesthetics and Cancer Biology: What Now? Where Are We Going?

**DOI:** 10.1002/adbi.202500122

**Published:** 2025-08-07

**Authors:** Eduardo Nunez‐Rodriguez, Hao Zhang, Dhananjay Sah, Juan P. Cata

**Affiliations:** ^1^ Department of Anesthesiology and Perioperative Medicine The University of Texas MD Anderson Cancer Center 1515 Holcombe Blvd. Houston TX 77030 USA; ^2^ Anesthesiology and Surgical Oncology Research Group The University of Texas MD Anderson Cancer Center 1515 Holcombe Blvd. Houston TX 77030 USA; ^3^ Department of Anesthesiology, Zhongshan Hospital Fudan University Shanghai 200031 China; ^4^ Shanghai Key Laboratory of Perioperative Stress and Protection Fudan University Shanghai 200031 China

**Keywords:** cancer, local anesthetics, metastasis

## Abstract

Local anesthetics (LAs), commonly  used for   regional and  general anesthesia, have gained attention in recent  years for their potential role during cancer curative surgery, as they may reduce cancer recurrence and progression. Studies in both laboratory and animal models have shown that LAs can inhibit tumor growth and cell proliferation, trigger apoptosis, and reduce metastasis by limiting cancer cell invasion and migration. In addition, LAs impact the tumor microenvironment by modulating inflammation, enhancing the immune response, blocking angiogenesis, and interfering with tumor innervation. The mechanisms behind these effects involve both voltage‐gated sodium channel‐dependent and independent pathways, such as AKT/mTOR, RAS/ERK, and SRC/STAT3, as well as regulating microRNAs, circular RNAs, and apoptosis‐related proteins, among others. Furthermore, LAs may enhance the efficacy of chemotherapy and counteract chemoresistance. The aim of this review is to provide a comprehensive summary of the current literature on the various mechanisms through which LAs influence tumorigenesis, alter metastasis processes, modulate immune responses, and affect angiogenesis within the tumor microenvironment.

## Graphical abstract

Local anesthetics (LAs), widely used in anesthesia, may also play a role in cancer treatment by inhibiting tumor growth, reducing metastasis, and enhancing immune responses. They modulate key pathways like AKT/mTOR and RAS/ERK while affecting angiogenesis and tumor innervation and the immune response. This review explores the mechanisms through which LAs influence cancer progression and therapy.

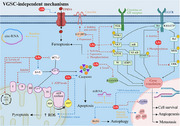

## 1 Introduction

Cancer remains a leading cause of morbidity and mortality worldwide, with lung, colorectal, breast, and prostate cancers among the most prevalent.^[^ ^1^, ^2^ ^]^ Despite the use of standard cancer treatments such as surgical resection, radiotherapy, chemotherapy, hormone therapy, immunotherapy – either individually or in combination – the risk of recurrence and disease progression remains high.^[^ ^3^ ^]^ For instance, up to 80% of patients who undergo surgical resection for pancreatic cancer experience recurrence within the first 26 months, and 30% recurring after 5 years, even when 97% had no evidence of metastasis before surgery and 90% had a clean surgical resection margin (R0).^[^ ^4^, ^5^ ^]^ In disease‐free lung cancer patients following primary treatment, approximately one‐third experience recurrence, with distant disease being the most common anatomical site of recurrence.^[^ ^6^ ^]^ As a result, there is an increasing interest in exploring new therapies to target mechanisms of cancer progression both perioperatively and beyond.^[^ ^7^ ^]^

Currently and historically, local anesthetics (LAs) have been used to provide local and regional anesthesia,^[^ ^8^ ^]^ blunt sympathetic responses to endotracheal intubation,^[^ ^9^ ^]^ and act as antiarrhythmic agents.^[^ ^10^ ^]^ A less well‐known effect is their activity against certain cancer cells. First described in the late 1970s, lidocaine was shown to improve the survival rates of mice with breast cancer by enhancing the tumor‐suppressing effects of hyperthermia. These initial results were attributed to lidocaine's influence on cell membrane fluidity.^[^ ^11^ ^]^ Subsequent studies indicated that LAs influence adenosine triphosphate (ATP) production. In particular, lidocaine and bupivacaine have been shown to reduce the viability of melanoma cells by impacting their metabolism and diminishing tumor energy, possibly due to glycolysis inhibition^[^ ^12^, ^13^ ^]^ and detachment of glycolytic enzymes from the cytoskeleton.^[^ ^13^ ^]^ More recent in vitro and in vivo experiments further support the anti‐tumor effects of LAs, including lidocaine, bupivacaine, and ropivacaine activity against colon,^[^ ^14^ ^]^ ovarian,^[^ ^15^ ^]^ breast,^[^ ^16^ ^]^ glioma,^[^ ^17^ ^]^ thyroid,^[^ ^18^ ^]^ hepatic,^[^ ^19^ ^]^ and prostate^[^ ^15^ ^]^ cancer cell lines, among others. In addition to the direct effects on malignant cells, LAs have anti‐angiogenic^[^ ^20^ ^]^ and immunomodulatory properties,^[^ ^21^ ^]^ even when administered at concentrations typically used in clinical settings.^[^ ^22^ ^]^

The objective of this review is to comprehensively summarize the current literature on the various mechanisms by which local anesthetics influence tumorigenesis, alter metastasis processes, modulate immune responses, and affect angiogenesis within the tumor microenvironment. While previous reviews have been primarily focused on the anticancer effects of systemic lidocaine and other LAs in the context of regional anesthesia, our work centers on the existing laboratory and clinical evidence regarding their impact when administered locally, including peritumoral infiltration or topically during oncological surgery.

## 2 Cell Proliferation and Survival

The analgesic and antinociceptive effects of LAs are linked to their action on voltage‐gated sodium channels (VGSCs), where they block inward sodium currents.^[^ ^23^ ^]^ The VGSC protein consists of α and β subunits. Nine α subunits have been identified (Nav1.1‐Nav1.9), and most are overexpressed in various cancer cell lines. Cancer cells that express functional VGSCs have a selective advantage in tumorigenesis and metastasis compared to cells that do not express these channels.^[^ ^24^ ^]^

**Figure 1:**
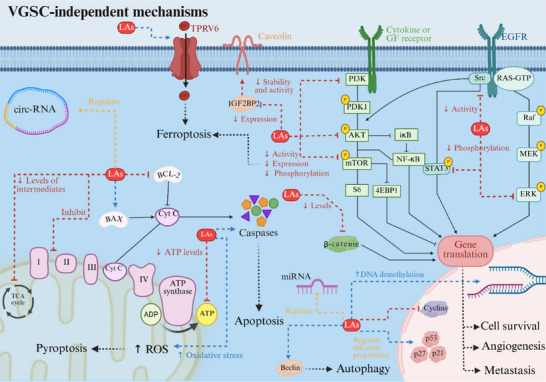
LAs affect both cancer cell proliferation and metastatic processes by multiple VGSC‐independent mechanisms. LAs’ antiproliferative role in cancer cells has been attributed to their impact on cell membrane receptors and multiple intracellular pathways, as well as their modulation of gene expression.

Experimental studies have revealed that LAs modify mechanisms involved in cell proliferation and survival by acting on VSGCs,^[^ ^25^ ^]^ impacting other membrane‐associated proteins^[^ ^26^ ^]^ and their intracellular pathways,^[^ ^26^ ^]^ and regulating gene expression.^[^ ^27^ ^]^ (**Figure** **1**).

### 2.1 Regulation of mTOR, AKT, and ERK Signaling

The mTOR signaling pathway involves both AKT and ERK, playing a role in protein synthesis, gene translation, cytoskeleton remodeling, and cell proliferation, migration, and metabolism.^[^ ^28^ ^]^ Amide LAs such as ropivacaine, bupivacaine, and lidocaine inhibit the AKT/mTOR axis by decreasing the activity of Akt‐associated kinase B,^[^ ^29^, ^30^ ^]^ and by reducing the expression^[^ ^27^, ^31^ ^]^ or phosphorylation^[^ ^32^, ^33^, ^34^, ^35^, ^36^, ^37^ ^]^ of PI3K, AKT, or mTOR, as well as other molecules involved in the pathway, including rS6 and 4EBP1,^[^ ^33^ ^]^ ultimately triggering autophagy.

Importantly, while some studies suggest these effects occur at clinically relevant concentrations (≈1 to 4 µg mL^−1^, depending on the route of administration),^[^ ^38^, ^39^ ^]^ others rely on concentrations far exceeding thresholds associated with neurological and cardiac toxicity (5–10 µg mL^−1^).^[^ ^40^, ^41^, ^42^, ^43^ ^]^ For instance, Zhang et al. utilized lidocaine concentrations ranging from 117 to 1874 µg mL^−1^ (0.5–8 mM) to demonstrate that it inhibits the expression of ubiquitin‐specific peptidase‐14, a protein positively associated with PI3K expression.^[^ ^37^ ^]^ This highlights the importance of critically appraising the experimental conditions and dosing strategies used in‐vitro.

Despite variability in the concentrations used across experimental settings, multiple mechanisms have been implicated in the ability of local anesthetics to modulate the AKT/mTOR pathway. In hepatocellular carcinoma cells, bupivacaine inhibited several pathway activators, including endothelial growth factor receptor (EGFR), ERBB3, MEK1, MEK2, KRAS, MAPK1, and BRAF, while increasing the expression of negative regulators such as PTEN, PP2A, and PHLPP1‐NF1.^[^ ^27^ ^]^ In breast cancer cells, ropivacaine formed a highly stable bond to AKT1 and reduced activity and inhibition of the NF‐κB signaling pathway, ultimately leading to the downregulation of GGT1 expression – an enzyme linked to poor prognosis in breast cancer.^[^ ^29^ ^]^ LAs also reduced NF‐κB signaling; through reducing IκB phosphorylation.^[^ ^44^ ^]^

Src, an activator of the AKT/mTOR pathway, is also targeted by LAs.^[^ ^45^ ^]^ Piegeler et al. incubated lung adenocarcinoma cells with increasing concentrations of lidocaine and ropivacaine and showed a 62% decrease in basal Src activity.^[^ ^34^, ^46^ ^]^ Notably, Piegeler's study utilized clinically relevant concentrations of lidocaine (2.88 µg mL^−1^).^[^ ^34^ ^]^ Ropivacaine also upregulated SNX10 expression, consequently inhibiting the Src/STAT3 signaling pathway, which led to a decrease in the proliferation of gastric cancer cells.^[^ ^47^ ^]^ Furthermore, ropivacaine inhibited the growth of cervical cancer cells by suppressing phosphorylated STAT3, which correlated with the downregulation of miR‐96 and MEG2.^[^ ^48^ ^]^

ERK abnormal upregulation is another pathway associated with cancer development. Experimental data revealed that lidocaine, bupivacaine, ropivacaine, or tetracaine cause a concentration‐dependent inhibition^[^ ^49^, ^50^ ^]^ and downregulation of ERK phosphorylation pathways in renal, melanoma, gastric, oral squamous, and colorectal cancer cells.^[^ ^51^, ^52^, ^53^, ^54^ ^]^ In thyroid cancer cells, lidocaine and bupivacaine at low millimolar concentrations demonstrated not only a time‐dependent downregulation of phosphorylated ERK but also caused disruption of mitochondrial integrity and an increased Bax/Bcl‐2 ratio, observed in apoptotic cells.^[^ ^17^ ^]^ Increased levels of pro‐apoptotic markers, such as Bax and caspases, along with decreased levels of anti‐apoptotic markers, like Bcl‐2, were also observed in hepatocellular carcinoma cells treated with lidocaine at clinically relevant dosages.^[^ ^55^ ^]^

Caveolin‐1 is an intrinsic membrane protein that plays a role in tumor cell growth and ERK signaling interaction. Lidocaine inhibited oral squamous cell proliferation and tumor growth by reducing the expression of insulin‐like growth factor 2 mRNA binding protein 2, which subsequently impaired caveolin‐1 stability and activity.^[^ ^25^ ^]^

In summary, LAs significantly influence cancer cell proliferation and survival by modulating various processes linked to key proteins and signaling pathways involved in gene expression regulation.

### 2.2 Mitochondria and Intracellular Metabolism

LAs may induce structural and functional changes in the mitochondria of cancer cells, such as swelling and ridge breakage, disruption of cell respiration, and triggering of oxidative stress.^[^ ^56^ ^]^ In breast cancer cells, ropivacaine and lidocaine at clinically relevant concentrations decreased mitochondrial respiration by inhibiting respiratory complexes I and II, leading to reduced levels of Krebs cycle intermediates, such as fumarate, malate, and alanine.^[^ ^33^, ^57^ ^]^ Levobupivacaine and bupivacaine have further shown to reduce ATP levels and increase reactive oxygen species (ROS) in prostate and neuroblastoma cancer cells, respectively.^[^ ^58^, ^59^ ^]^ Furthermore, lidocaine (0.5 mM) lowered the levels of glutathione and its precursor, glutamate, which suggests heightened consumption in response to oxidative stress in breast cancer cells.^[^ ^57^ ^]^ Similar results were shown by Okamoto et al. in renal carcinoma cells expressing von Hippel‐Lindau protein. Interestingly, the loss of hypoxia‐inducible factor 1 reversed lidocaine's effect.^[^ ^60^ ^]^

LAs also impact on cancer cell survival by regulating mitochondrial‐circular RNA.^[^ ^61^, ^62^ ^]^ For instance, lidocaine decreased the viability and proliferation of colorectal cancer cells by inducing the overexpression of circ‐BANP and circ‐ITFG2, consequently affecting the SOCS2 axis and downregulation of circ‐DDX17.^[^ ^61^, ^63^ ^]^ Lidocaine administration also resulted in reduced tumor growth in a mouse model by decreasing the expression of circ‐PDZD8, which led to lower levels of Golgi transport 1A protein.^[^ ^64^ ^]^ This mechanism explained the inhibitory effects of lidocaine on lung cancer cells’ proliferation.^[^ ^65^ ^]^

Glycogen synthase kinase 3β is an enzyme involved in glucose metabolism and serves as a positive regulator of pathways that contribute to tumorigenesis. In ovarian and prostate cancer cells, bupivacaine at clinically relevant concentrations triggered apoptosis by inhibiting glycogen synthase kinase 3β phosphorylation.^[^ ^15^ ^]^ Yoo et al. revealed that intravesical instillation of lidocaine at a low millimolar concentration induced cancer cell autophagy and reduced tumorigenesis through glycogen synthase kinase 3β phosphorylation in a murine model of bladder cancer.^[^ ^66^ ^]^

In summary, LAs hinder cancer cell growth by elevating oxidative stress, modifying the expression of circular RNA, and overseeing processes related to ATP production, including oxidative phosphorylation and the Krebs cycle.

### 2.3 Regulation of Cell Cycle Progression

Cell cycle progression is heavily reliant on the interaction and signaling of proteins that serve as regulators or checkpoints, such as cyclins, p proteins, and beclin‐1. In melanoma cells, ropivacaine and levobupivacaine at low micromolar concentrations reduced cancer cell viability and proliferation by lowering cyclin D and E while increasing p21, p27, and the active form of p53.^[^ ^67^ ^]^ Similarly, in bladder cancer cells, ropivacaine decreased p62 levels and increased beclin‐1, which correlated with the induction of autophagy.^[^ ^31^ ^]^ Izdebska et al. further reported heightened beclin‐1 expression and autophagy when glioma cells were treated with millimolar concentrations of lidocaine.^[^ ^68^ ^]^ In this study, lidocaine promoted in a dose‐dependent manner (0.25 30 mM) the formation of vacuole‐like structures surrounded by vimentin, indicating reorganization of the cytoskeleton.

### 2.4 Regulation of Ferroptosis and Pyroptosis

LAs have also shown to regulate ferroptosis, a non‐apoptotic cell death pathway resulting from the accumulation of iron‐dependent lipid peroxides.^[^ ^69^ ^]^ In both ovarian and breast cancer cells, ropivacaine and lidocaine triggered ferroptosis by inhibiting PI3K/AKT and reducing GPX4 and SCL7A11 levels, respectively.^[^ ^70^, ^71^ ^]^ Similarly, glioblastoma cells exposed to 2–8 millimolar concentrations of lidocaine exhibited increased cytosolic calcium concentrations and transient receptor potental cation channel subfamily V member 1 phosphorylation through CaMKII, leading to pyroptosis, an inflammatory programmed cell death pathway characterized by the release of inflammatory mediators and ROS.^[^ ^72^ ^]^

### 2.5 Epigenetic Regulation, Gene Translation and Gene Transcription

Regulation of epigenetic and gene expression processes are additional mechanisms through which LAs influence tumorigenesis. In breast cancer cells, lidocaine and ropivacaine at clinically relevant concentrations increased the expression of tumor suppressor genes by demethylating DNA.^[^ ^73^ ^]^ Additionally, lidocaine modulated cytoplasmic RNA polyadenylation by upregulation of cytoplasmic polyadenylation element binding protein 3 in hepatocellular carcinoma cells.^[^ ^74^ ^]^ LAs also regulated the Wnt/β‐catenin pathway, where β‐catenin translocated to the nucleus to activate targeted gene transcription. Ropivacaine, lidocaine, and bupivacaine reduced leukemia stem cell proliferation by lowering β‐catenin levels and upregulating Axin, a known negative regulator of this pathway.^[^ ^75^ ^]^ These effects were observed in a dose‐dependent manner and at clinically relevant concentrations.

In bladder cancer cells, lidocaine inhibits proliferation by downregulating the expression of isoprenylcysteine carboxylmethyltransferase,^[^ ^76^ ^]^ an enzyme involved in the posttranslational prenylation processing pathway of oncogenic proteins, including RAS – which is crucial to the stability and proper function of many carcinogenic proteins.^[^ ^77^ ^]^ LAs have shown to impair the growth of glioblastoma stem cells by depleting ZDHHC15 and consequently decreasing protein s‐palmitate esterification, another pathway of post‐translational modification.^[^ ^78^ ^]^

Experimental in vitro studies demonstrate that LAs modulate gene expression by modulating microRNAs (miRNAs). For example, lidocaine inhibited retinoblastoma cells proliferation by increasing miR‐520a‐3p, leading to decreased levels of EGFR.^[^ ^79^ ^]^ In cervical cancer cells, lidocaine reduced cell viability in a time‐dependent and a dose‐dependent manner by modulating the miR‐421/BTG1 signaling pathway.^[^ ^80^ ^]^ Similarly, lidocaine at low millimolar concentrations caused downregulation of miR‐21‐5p and upregulation of LIFR, a known cancer suppressor,^[^ ^81^ ^]^ resulting in the inhibition of cell proliferation.^[^ ^82^ ^]^ Ropivacaine and lidocaine reduced glioma cell proliferation by regulating circSCAF11/miR‐145‐5p and circEZH2/miR‐181b‐5p, respectively.^[^ ^56^, ^83^ ^]^ In breast cancer cells, ropivacaine diminished tumor growth by modulating the miR‐27b‐3p/YAP pathway axis.^[^ ^84^ ^]^ Additionally, ropivacaine inhibited the proliferation of glioblastoma cells by targeting the same miRNA (miR21‐5p) and KANSL2 gene.^[^ ^85^ ^]^ Finally, in hepatocellular carcinoma cells, lidocaine at clinically relevant concentrations inhibited proliferation in a dose‐dependent manner by regulating the circ_ITCH/miR‐421 pathway, which resulted in increased levels of cytoplasmic polyadenylation element binding protein 3.^[^ ^86^ ^]^

The endoplasmic reticulum is an organelle associated with the anticancer effects of LAs. Treating neuroblastoma cells with lidocaine results in an increased apoptotic rate due to a rise in both the mRNA and protein levels of binding immunoglobulin protein, PKR‐like ER kinase, activating transcription factor 4, and CCAAT/enhancer‐binding protein homologous protein, which are known endoplasmic reticulum stress–associated molecules.^[^ ^87^ ^]^

Overall, LAs influence gene expression in cancer cells by modulating transcription and translation processes. This includes DNA demethylation, downregulation of the Wnt/β‐catenin pathway, activity of posttranslational enzymes, and endoplasmic reticulum functions, along with the regulation of miRNAs signaling.

## 3 Invasion and Metastasis

**Figure 2:**
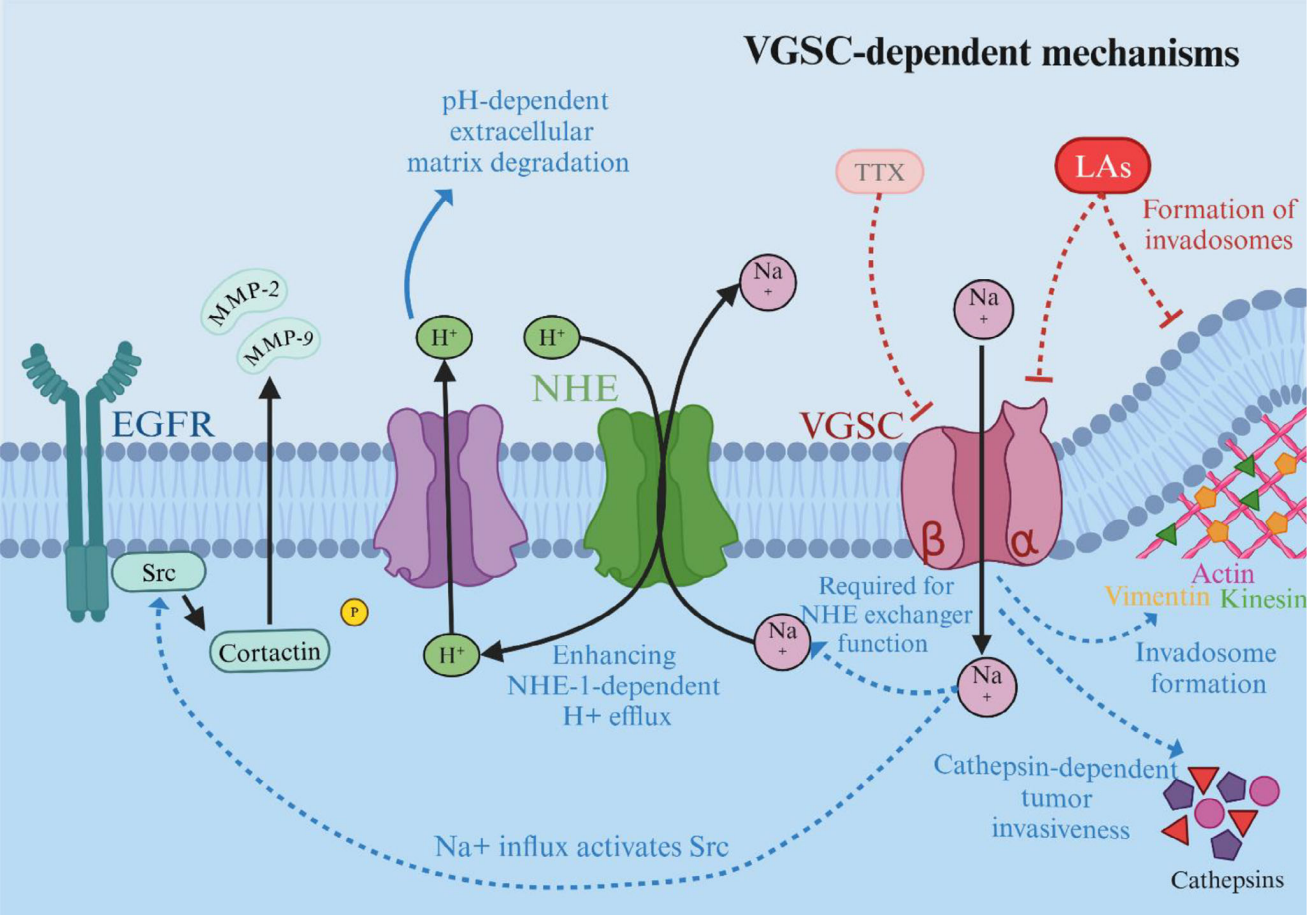
VGSC‐dependent mechanisms affect cancer cell invasion and metastasis. By inhibiting Nav channels, LAs are thought to decrease VGSC‐induced NHE exchanger function, which promotes pH‐dependent extracellular matrix degradation, the EGFR/Src/cortactin/MMP pathway, cathepsin‐dependent tumor invasiveness, and invadosome formation.

Metastasis is a hallmark of cancer and remains the leading cause of cancer‐related deaths. The metastatic process involves several steps: cancer cells must leave their primary site, circulate through the bloodstream, survive within blood vessels, establish themselves at a secondary site, and evade immune cells. LAs may influence all of these steps through various mechanisms involving both VGSC‐independent^[^ ^53^, ^82^, ^88^, ^89^, ^90^ ^]^ (Figure 1) and VGSC‐dependent signaling.^[^ ^91^, ^92^ ^]^ (**Figure** **2**)

### 3.1 Epithelial‐Mesenchymal Transition

Epithelial‐mesenchymal transition (EMT) represents the initial phase of metastasis, during which epithelial cells acquire the invasive characteristics of mesenchymal cells.^[^ ^93^, ^94^ ^]^ Lidocaine reduced the expression of mesenchymal markers, such as N‐cadherin and vimentin, while increasing the expression of the epithelial marker E‐cadherin. Additionally, lidocaine at low millimolar concentrations decreased the mRNA expression and enzymatic activity of metalloproteinases 2 and 9, which are known for their key role in cell invasion.^[^ ^92^, ^95^ ^]^ In gastric cancer cells, lidocaine induced downregulation of Snail, a molecule recognized for promoting EMT.^[^ ^82^ ^]^

### 3.2 VGSC‐Dependent Mechanisms

VGSC activity drives cellular migration and invasion in different cancer cell lines.^[^ ^96^, ^97^, ^98^ ^]^ Evidence indicates a positive correlation between the expression levels of VGSCs and the ability of cancer cells to invade the basement membrane.^[^ ^99^ ^]^ In highly metastatic cells, the inhibition of VGSCs significantly reduced cells’ capacity for invasion.^[^ ^97^, ^100^ ^]^ Gillet et al. demonstrated that the invasiveness of breast cancer cells was significantly reduced in the presence of tetrodotoxin.^[^ ^98^ ^]^ The authors proposed that the link between VGSC activity and invasion was mediated through the modulation of cathepsin activity. Similarly, Baptista‐Hon et al. reported that tetrodotoxin impaired the invasion of colon cancer cells. In these and other studies, Nav1.5 was identified as the primary target channel.^[^ ^91^, ^98^, ^101^ ^]^ In a randomized clinical trial involving lung cancer patients, perioperative intravenous infusion of lidocaine led to significantly fewer EMT biomarkers and tumor metastasis.^[^ ^102^ ^]^ This finding contrasts with results from in vitro studies suggesting that levobupivacaine and lidocaine induce EMT.^[^ ^103^, ^104^ ^]^

Invadopodia and podosomes, collectively referred to as “invadosomes,” are actin‐based structures enriched with VGSCs and strongly associated with tumor invasive capacity.^[^ ^105^ ^]^ Cellular acidification in the invadopodia region of the plasma membrane is a key mechanism of cell invasion. Acidification occurs as Na⁺ enters through VGSCs, facilitating the activity of the Na⁺/H⁺ exchanger, which imports H⁺ ions and enhances Na⁺/H⁺ exchanger–dependent H⁺ efflux. This process is essential for the pH‐dependent degradation of the extracellular matrix in invadopodia, thereby promoting tumor invasion.^[^ ^106^ ^]^ Beyond invadosomes, highly metastatic tumor cell lines utilize microtentacles for invasion with support of vimentin filaments.^[^ ^107^ ^]^ Studies have shown that lidocaine and tetracaine at clinically non‐toxic concentrations inhibited the extension of microtentacles and the activity of molecules involved in cell migration, such as vimentin and kinesin.^[^ ^108^ ^]^

Lastly, VGSCs are believed to promote invasion and metastasis by operating within the EGFR/Src/cortactin signaling axis. Sodium influx through Nav1.5 channels activates Src, which phosphorylates cortactin, leading to the upregulation and secretion of MMP‐2 and MMP‐9. This process results in the degradation of the extracellular matrix.^[^ ^106^, ^109^ ^]^ In a study by Brisson et al., VGSCs were also shown to alter actin polymerization and enhance the invasive morphology of cancer cells.^[^ ^88^, ^89^ ^]^

In conclusion, VGSCs are intricately involved in intracellular processes that facilitate metastasis, such as invadosome formation, pH‐dependent breakdown of the extracellular matrix, and the release of metalloproteinases. By blocking these channels LAs could influence cancer cell invasion and migration.

### 3.3 VSGC‐Independent Mechanisms

The CXCR4 receptor promotes cell invasion by regulating calcium metabolism.^[^ ^110^ ^]^ Lidocaine has been shown to reduce cell migration in non‐squamous lung and breast cancer cells by inhibiting CXCL12‐CXCR4 signaling, thereby reducing calcium‐dependent cytoskeleton remodeling.^[^ ^88^ ^]^ Similarly, in breast, prostate, and ovarian cancer cells, lidocaine (>2 mM) decreased invasion and migration by downregulating the TRPV6 receptor, which led to a subsequent reduction in calcium influx.^[^ ^111^ ^]^

As previously discussed, LAs regulate several signaling pathways and molecular mechanisms, including PI3K/AKT/mTOR, MAPK,^[^ ^27^, ^29^, ^30^, ^31^, ^36^, ^37^ ^]^ ERK,^[^ ^50^ ^]^ caveolin‐1,^[^ ^112^ ^]^ circular RNAs,^[^ ^71^, ^82^, ^83^, ^84^, ^85^, ^86^, ^113^ ^]^ and miRNAs.^[^ ^56^, ^114^ ^]^ They also influence mitochondrial function,^[^ ^63^, ^64^, ^83^, ^86^ ^]^ oxidative damage, and cell membrane receptors, such as ITGB1,^[^ ^53^ ^]^ TASK‐3,^[^ ^115^ ^]^ and ITGA2.^[^ ^115^ ^]^ These signaling pathways play a crucial role in migration and invasion, processes essential for metastasis. For example, the incubation of papillary thyroid cancer cells with low micromolar concentrations of ropivacaine significantly inhibited cell migration and MMP‐9 expression, an effect attributed to the suppression of ITGA2 expression.^[^ ^115^ ^]^

Paxillin is a protein essential for focal adhesions, which mediate connections between the extracellular matrix and the cytoskeleton and are crucial for integrin receptor signal transduction.^[^ ^116^ ^]^ In esophageal cancer cells, ropivacaine at low concentrations reduced migration through prenylation‐dependent inhibition of the FAK/paxillin pathway.^[^ ^90^ ^]^ Similarly, in colon cancer cells, ropivacaine inhibited migration by reducing integrin β1 expression and modulating the activation of its downstream signaling pathways.^[^ ^53^ ^]^

In vivo studies support the antimetastatic properties of LAs. Johnson et al. investigated the effects of lidocaine using a breast tumor resection mouse model, that partially mimicked the formation of distant metastases after surgery. Lidocaine was administered intravenously as a 1.5 mg kg^−1^ bolus via a tail‐vein cannula, followed by a continuous infusion of 2 mg kg h^−1^ for 25 min. When combined with sevoflurane anesthesia, intravenous lidocaine significantly reduced the pulmonary metastatic burden.^[^ ^117^ ^]^ In another study, intraperitoneal administration of lidocaine improved survival in mice with peritoneal carcinomatosis.^[^ ^118^ ^]^ More recently, lidocaine (30 mg kg^−1^) was administered five times per week for 23 days in an orthotopic murine model of breast cancer surgery; significantly reducing pulmonary metastatic disease in animals treated with either propofol or sevoflurane as the primary anesthetic during surgical resection.^[^ ^119^ ^]^

In summary, LAs reduce cancer cell metastasis by influencing VGSC‐independent pathways. This encompasses the modulation of membrane receptor signaling, involving CXCR4, ITGB1, TASK‐3, and ITGA2, along with intracellular pathways linked to reduced cancer cell proliferation, such as mTOR/AKT, ERK, caveolin, miRNA, and circular RNA signaling, among others.

## 4 Combination with Other Chemotherapeutic Agents and Other Cancer Treatments

Chemotherapy remains the cornerstone of treatment for various malignancies. Preclinical evidence suggests that LAs can synergistically enhance the efficacy of chemotherapy agents^[^ ^55^, ^120^, ^121^ ^]^ or help alleviate chemoresistance.^[^ ^122^ ^]^ Several mechanisms underlying these effects have been identified (**Table** **1**). For example, lidocaine reduced temozolomide resistance in glioblastoma multiforme cells by modulating the mesenchymal‐epithelial transition factor.^[^ ^123^ ^]^ It also diminished cisplatin resistance in ovarian cancer cells by inhibiting Nav1.5 and blocking the FAK/paxillin pathway.^[^ ^92^ ^]^ Additionally, lidocaine reversed cisplatin resistance in cutaneous squamous cell carcinoma through the upregulation of miR‐30c and the downregulation of SIRT1.^[^ ^124^ ^]^ In breast cancer cells, lidocaine at toxic clinically concentrations induced global DNA demethylation and upregulated the expression of RARβ2 and RASSF1A, thereby sensitizing the cells to cisplatin.^[^ ^125^ ^]^ Furthermore, lidocaine, along with ropivacaine and bupivacaine, in low millimolar concentrations potentiated the cytotoxic effects of cisplatin in hepatocarcinoma cells by increasing RASSF1A expression.^[^ ^126^ ^]^

**Table 1:** Enhancing effect of LAs on chemotherapeutic drugs.

Author [Year]	Chemotherapeutic drug [concentration]	Cell line	Cancer histology	Local Anesthetic [concentration]	In vitro experiment	Mechanism	In vivo experiment
Xing, et al. [2017]	Cisplatin [10 µM]	HepG2	Hepatocellular carcinoma	Lidocaine [5 mM]	↓ cell viability and ↑ apoptosis	↑ activity of Bax and caspase‐3↓ levels of Bcl‐2	↓ tumor weight
Liu, et al. [2021]	Cisplatin [10 µM]	SKOV3, A2780, and ID8	Ovarian adenocarcinoma	Lidocaine [5 mM]	↓ cell viability and ↑ apoptosis	Blocking of Nav1.5 mediated FAK/Paxillin pathway↑ expression of cleaved PARP, caspase‐3, and caspase‐8↓ levels of Bcl‐2	↓ tumor weight
Zhang, et al. [2020]	Cisplatin [30 µM]	MGC‐803/DDP	Gastric adenocarcinoma	Lidocaine [200 µM]	↓ cell viability and ↑ apoptosis	↓ levels of miR‐10b, p‐AKT and p‐mTOR, and β‐catenin ↑ levels of Bax, cleaved‐caspase‐3 and cleaved‐PARP protein	Not performed
Liu, et al. [2022]	Cisplatin [50 µM]	A431 or A431‐R	Cutaneous squamous cell carcinoma	Lidocaine [10 mM]	↓ cell proliferation	↑ levels of miR‐30c↓ levels of SIRT1	Not performed
Li, et al. [2014]	Cisplatin [0.2 µM]	MCF‐7 and MDA‐MB‐231	Breast	Lidocaine [0.1 mM]	↓ cell clone number and cell proliferation and ↑ apoptosis	↓ levels of RARβ2 or RASSF1A↑ levels of procaspase 3, PARPPARP, and cytochrome c	Not performed
Chen, et al. [2020]	Cisplatin [10 µM]	HepG2 and BEL‐7402	Hepatocellular carcinoma	Lidocaine, ropivacaine, and bupivacaine [0.5 mM]	↓ cell number and ↑ cytotoxicity	↑ RASSF1A expression	Not performed
Zhang, et al. [2019]	5‐Fluorouracil [10 and 100 µM]	JEG‐3 and JAR	Choriocarcinoma	Lidocaine [1, 10, or 100 µM]	↓ cell viability and ↑ apoptosis	↓ levels of ABCG2↓ levels of p‐PI3K and p‐AKT	Not performed
Wang, et al. [2017]	5‐Fluorouracil [1, 10, and 100 µM]	SK‐MEL‐2	Melanoma	Lidocaine [10 and 100 µM]	↓ cell viability and ↑ apoptosis	↑ Bax, cleaved caspase‐3, and cleaved caspase‐9↓ Bcl2↑ expression of miR‐493, leading to ↓ Sox4 expression and consequently inactivation of PI3K/AKT	Not performed
Wang, et al. [2024]	Sorafenib [0.5 µM]	HepG2 and Huh7	Hepatocellular carcinoma	Ropivacaine [20 µM]	↓ cell viability	↓ expression of miR‐224↑ increased HOXD10 expression	Not performed
Wang, et al. [2024]	Sorafenib [0 to 31.25 µM]	HepG2 and Huh7	Hepatocellular carcinoma	Ropivacaine [0 to 1000 µM]	↓ cell proliferation and growth↑ apoptosis	↑ expression of cleaved caspase‐3, cleaved caspase‐9, and cyclinD1↓ expression of IL‐6 and p‐STAT3	↓ tumor volume and weight
Seok, et al. [2022]	Palbociclib [0.1 and 0.5 µM]	MDA‐MB 231, MDA‐MB 453, and 4T1	Breast	Lidocaine [2 and 3 mM]	↓ cell proliferation, migration, and invasion↑ apoptosis	↓ expression of Rb, E2F1, cyclin B1, p‐AKT, p‐GSK3β, and E‐cadherin↑ release of cytochrome c, activation of cleaved‐caspase3, and vimentin	↓ tumor volume
Chen, et al. [2024]	Temozolomide [125 and 250 µM]	U87MG and DBTRG‐05MG	Glioblastoma	Lidocaine [0.75 mM]	↓ cell viability and migration	↓ expression and activation of MET	Not performed

Palbociclib is a kinase inhibitor that targets the cell cycle machinery.^[^ ^127^ ^]^ Lidocaine has been shown to enhance the effects of palbociclib in triple‐negative breast cancer cells, leading to increased cell cycle arrest, reduced retinoblastoma protein levels, diminished PI3K/AKT signaling, and enhanced apoptosis.^[^ ^128^ ^]^ Sorafenib, a multispecific inhibitor targeting multiple kinases, has also been studied in combination with LAs.^[^ ^129^ ^]^ Ropivacaine enhanced the activity of sorafenib against hepatocellular carcinoma cells by inhibiting miR‐224 expression and targeting HOXD10.^[^ ^130^ ^]^ Furthermore, the combination of ropivacaine and sorafenib synergistically suppressed the interleukin (IL)‐6/STAT pathway, hindering the viability and proliferation capacity of hepatocellular carcinoma cells.^[^ ^131^ ^]^

A nanostructured lipid carrier hydrogel containing a combination of ropivacaine and doxorubicin inhibited tumor progression after surgical excision in a mouse model of locally recurrent melanoma. Nanostructured lipid carriers co‐loaded with docetaxel and lidocaine also led to decreased melanoma cell viability and growth.^[^ ^132^ ^]^ In a mouse model of hepatocellular carcinoma, lidocaine potentiated the effects of cisplatin by increasing the Bax/Bcl‐2 ratio and caspase levels.^[^ ^55^ ^]^ Furthermore, a complex formed by lidocaine and 2‐hydroxypropyl‐β‐cyclodextrin significantly reduced the viability of oral squamous cell carcinoma cells.^[^ ^133^ ^]^

Hyperthermia is a recognized mechanism for inducing cell death. Lidocaine has been shown to enhance thermal damage in melanoma, cervical cancer, and basal cell carcinoma in a dose‐dependent manner. Raff et al. demonstrated that cancer cell lines exhibit significantly increased cell toxicity when lidocaine concentrations of 0.1% to 0.2% are combined with hyperthermia at 42 °C.^[^ ^134^ ^]^ Photodynamic therapy utilizes a light‐activated drug and a light source to destroy cancer cells.^[^ ^135^ ^]^ In a mouse study, photofrin was employed as a photosensitizer and vasodilator. The combination of lidocaine and photofrin resulted in decreased tumor growth compared to photofrin alone.^[^ ^136^ ^]^

In conclusion, preclinical studies suggest that LAs may enhance the efficacy of existing cancer therapies, including chemotherapy (e.g., temozolomide, cisplatin, doxorubicin, palbociclib, and sorafenib), as well as photodynamic therapy and hyperthermia.

## 5 Tumor Microenvironment

### 5.1 Inflammation

Inflammation and immune suppression are key features of cancer. Both preclinical and clinical studies have demonstrated that LAs possess significant anti‐inflammatory effects.^[^ ^21^, ^137^, ^138^ ^]^ Neutrophils play a central role in inflammation and are involved in various aspects of tumor growth and metastasis.^[^ ^139^ ^]^ LAs inhibit not only neutrophil priming and superoxide anion production but also their mobility.^[^ ^140^ ^]^ Specifically, lidocaine has been demonstrated to reduce the chemo‐attractive and metabolic effects induced by lysophosphatidic acid in primed human neutrophils.^[^ ^141^ ^]^ Research by Sixt et al. revealed that exposure to increasing concentrations of bupivacaine, levobupivacaine, and lidocaine resulted in reduced neutrophil migration for all three LAs.^[^ ^142^ ^]^ Piegeler et al. have shown that lidocaine and ropivacaine at clinically relevant concentrations reduced tumor necrosis factor (TNF‐α)‐induced activation of AKT, FAK, and caveolin‐1 in lung adenocarcinoma cells.^[^ ^34^ ^]^

Neutrophil extracellular traps (NETs) play a role in fostering a pro‐metastatic environment.^[^ ^139^ ^]^ In vitro research suggested that low concentrations of lidocaine (0.007–1.42 mmol L^−1^) enhanced NET formation, whereas high concentrations (≥7.1 mmol L^−1^) resulted in decreased formation.^[^ ^138^, ^143^, ^144^ ^]^ Additionally, lidocaine administered intravenously in infusion was shown to reduce NET formation and myeloperoxidase levels in patients undergoing various cancer surgeries. Nevertheless, these beneficial effects did not lead to better oncological outcomes, as indicated in a large clinical trial involving patients with resectable pancreatic carcinoma.^[^ ^143^ ^]^

Tumor‐associated macrophages play a significant role in the tumor microenvironment, contributing to tumor progression and metastasis through the promotion of inflammation. Research indicates that macrophages treated with lidocaine exhibit reduced proliferation and a decrease in the levels of inflammatory molecule activity following lipopolysaccharide treatment.^[^ ^145^ ^]^ This reduction correlates with the inhibition of HIF1α‐induced glycolysis. In a study by Fu et al., a hydrogel system was engineered to silence STAT3 and deliver lidocaine, which was subsequently tested in a mouse model of lung cancer. The results revealed that when lidocaine was encapsulated within endoplasmic reticulum–modified liposomes, there was a notable decrease in the proportion of M2 macrophages present in the tumor microenvironment.^[^ ^146^ ^]^ Furthermore, lidocaine was found to impede the production of pro‐inflammatory cytokines, including TNF‐α, IL‐6, and IL‐12 from dendritic cells. These findings are supported by clinical studies that demonstrate lidocaine effectively reduces plasma levels of IL‐6, IL‐8, complement C3a, and IL‐1ra in patients undergoing colorectal surgery.^[^ ^147^ ^]^

In summary, preclinical research indicates that LAs may influence cancer cell biology by attenuating inflammation. Although clinical studies demonstrate that LA compounds significantly decrease plasma concentrations of inflammatory cytokines, neutrophil extracellular trap formation, and myeloperoxidase levels, these effects did not yield improved clinical outcomes.

### 5.2 Immunity

Lymphocytes play a pivotal role in the immune system's response to cancer. These cells express VGSCs, which regulate their ability to traffic within the body.^[^ ^148^ ^]^ Fraser et al. conducted in vitro experiments utilizing Jurkat cells and demonstrated through patch clamp techniques that tetrodotoxin suppressed inward sodium currents in a dose‐dependent manner.^[^ ^148^ ^]^ Furthermore, they discovered that Jurkat cells treated with tetrodotoxin were less invasive than untreated cells. LAs may inhibit T‐cell proliferation and suppress important functions in T cells, such as IL‐2, and interferon gamma (IFN‐γ) secretion.^[^ ^149^ ^]^ Decreased levels of lymphocytes‐derived cytokines, such as IFN‐γ, IL‐2, IL‐4, IL‐10, and IL‐17, were observed in mice treated with lidocaine.^[^ ^117^ ^]^ T helper 17 cells represent a distinct subset of T helper lymphocytes, recognized for their significant production of IL‐17.^[^ ^150^ ^]^ This cytokine is implicated in tumor growth and metastasis. In a clinical study, serum levels of IL‐17 were found to be diminished were found to be diminished following lidocaine intravenous infusion in patients with lung cancer undergoing video‐assisted thoracoscopic surgery.^[^ ^151^ ^]^

The expression of major histocompatibility complex‐I on the surface of cancer cells is also pivotal in determining the effectiveness of anti‐tumor immune responses mediated by cytotoxic CD8+ lymphocytes.^[^ ^152^, ^153^ ^]^ Ropivacaine‐loaded hydrogels have demonstrated an ability to enhance major histocompatibility complex‐I expression in breast cancer cells, which has been correlated with a reduction in cancer recurrence in mouse models.^[^ ^154^ ^]^ Moreover, a lidocaine‐based hydrogel was found to increase the count of CD8+ lymphocytes within tumor tissues, reduce the number of regulatory T cells, and elevate serum levels ofIFN‐γ and TNF‐α. The recruitment of CD8+ lymphocytes was linked to enhanced activation of dendritic cells, which is essential for initiating anti‐tumor immunity in the draining lymph nodes associated with tumors.^[^ ^146^ ^]^

PD‐1 is a receptor located on the surface of T cells. serving as an “off switch” that inhibits T cells from recognizing cancer cells after they bind to PD‐L1.^[^ ^155^ ^]^ A PD‐1 inhibitor is a molecule that blocks this immune checkpoint protein. Bezu et al. conducted a study examining the effects of LAs in combination with PD‐1 inhibitors in murine models, discovering that ropivacaine and lidocaine exhibited additive effects.^[^ ^156^ ^]^ The authors proposed that the intratumoral administration of a LA could transform an immunologically “cold” tumor into a “hot” tumor, thereby promoting the recruitment of immune effectors.

Natural killer (NK) cells are a subset of lymphocytes and immune effectors that are integral to the initiation of innate immune responses against neoplastic cells. Numerous laboratories have investigated the effects of LAs on NK cell functionality, yielding mixed results. Initial in vitro studies indicated that LAs, such as lidocaine and procaine, diminished NK cell activity.^[^ ^157^ ^]^ However, more recent evidence indicates that lidocaine and bupivacaine may indeed enhance their cytolytic capabilities,^[^ ^158^ ^]^ even when utilizing clinically relevant concentrations (0.01 µM).^[^ ^21^ ^]^ When lidocaine is administered alongside sevoflurane, there appears to be a synergistic effect that inhibits NK cell function.^[^ ^117^ ^]^ In a clinical study, women undergoing surgical procedures for breast cancer who received an intravenous lidocaine infusion exhibited a greater percentage of NK cells compared to those who received a placebo.^[^ ^159^ ^]^ Conversely, a notable reduction in the proportion of NK cells was recorded following an epidural block using lidocaine, implying that the efficacy of local anesthetics is contingent upon the site of administration.^[^ ^160^ ^]^

Cancer‐associated fibroblasts (CAFs) play a central role in the tumor microenvironment by significantly influencing the behavior of cancer cells and other stromal cells.^[^ ^161^ ^]^ CAFs facilitate tumorigenic pathways, induce EMT, stimulate cancer cell invasion, and trigger angiogenesis. LAs have notable effects on fibroblasts, including the inhibition of their proliferation. Initially, it was postulated that this inhibitory effect was due to changes in membrane potential.^[^ ^162^, ^163^ ^]^ However, it was subsequently determined that this effect is related to the induction of reactive oxygen species (ROS).^[^ ^164^ ^]^ CAFs serve as key regulators of angiogenesis by secreting stromal cell–derived factor 1, which interacts with the CXCR4 receptor.^[^ ^165^ ^]^ Notably, lidocaine at clinically relevant concentrations has been shown to reduce the expression of CXCR4 and its associated signaling pathways.^[^ ^89^ ^]^ Experimental findings by Xing et al. demonstrated that this effect was linked to the ability of LAs to inhibit the migration of lung cancer cells.^[^ ^88^ ^]^

Mesenchymal stem cells are recognized for their immunomodulatory properties and capacity to target tumors.^[^ ^166^ ^]^ In vitro studies have shown that amide LAs can decrease cell viability and induce apoptosis in human mesenchymal stem cells.^[^ ^167^ ^]^ This effect transpires through a rapid reduction in intracellular calcium levels at the endoplasmic reticulum, triggering the apoptotic cell death cascade. More recent research conducted by Lucchinetti et al. has revealed that ropivacaine, at non‐clinically toxic dosages inhibits cell proliferation and triggers apoptosis in a dose‐dependent manner.^[^ ^168^ ^]^ Additionally, their findings indicated that ropivacaine reduces TNF‐α‐induced cell migration and ICAM expression, which are associated with decreased ATP production and increased ROS production.

In conclusion, LAs can boost anti‐tumor immunity by not only influencing stromal cell activity and proliferation but also by regulating the functions and cytokine production of NK cells, dendritic cells, and T cells.

### 5.3 Angiogenesis

Angiogenesis refers to the process through which new blood vessels are formed from preexisting vessels, involving the migration, proliferation, and differentiation of endothelial cells.^[^ ^169^ ^]^ This process is crucial for tumor development and metastasis. VGSCs, such as Nav1.5 and Nav1.7, are expressed in endothelial cells and are implicated in various angiogenic functions, including vascular endothelial growth factor (VEGF)–induced cell proliferation and chemotaxis. VGSCs have been proposed as a novel target for the regulation of angiogenesis.^[^ ^170^ ^]^

VEGF is considered one of the most important factors in tumor angiogenesis.^[^ ^171^ ^]^ Lidocaine has demonstrated the ability to inhibit angiogenesis, along with suppressing VEGF‐induced endothelial cell migration and proliferation. Furthermore, it induces apoptosis in endothelial cells via inhibition of VEGF‐induced phosphorylation of VEGF receptors, and interrupts signaling pathways associated with phospholipase C γ, protein kinase C, MAPK, and FAK‐paxillin in endothelial cells.^[^ ^20^, ^172^ ^]^ Noteworthy, this effect was described using clinically relevant concentrations (0.05–0.2 mM).^[^ ^20^ ^]^

Ropivacaine inhibits the formation, growth, and survival of tumor‐associated endothelial cells and their capillary networks by adversely affecting mitochondrial function and inducing oxidative stress.^[^ ^173^ ^]^ Bupivacaine similarly impairs angiogenesis by interfering with mitochondrial respiration. Additionally, its ability to inhibit the AKT/mTOR pathway and activate the AMPK pathway has been documented.^[^ ^174^ ^]^ ICAM‐1 plays a role in the adhesion and migration of endothelial cells, involving the VEGF and AKT/PI3K signaling pathways, and its expression decreases following treatment with lidocaine.^[^ ^175^, ^176^ ^]^

### 5.4 Tumor Neuroregulation

Tumor innervation plays a pivotal role in the progression and metastasis of cancer. Recent evidence indicates that the efficacy of anti‐PD‐1 treatment is significantly compromised when nerve damage is induced prior to administration in a murine model.^[^ ^177^ ^]^ Lidocaine has shown the capability to effectively inhibit tumor growth and nerve formation by downregulating the expression of nerve growth factor and neuronatin in a mouse model of breast cancer.^[^ ^178^ ^]^ Furthermore, bupivacaine has shown to reduce the viability of neuronal cells in the presence of 4T1 breast cancer cells.^[^ ^179^ ^]^ When administered in nanoparticles, bupivacaine inhibited tumor growth and metastatic dissemination in orthotopic breast cancer tumors.^[^ ^179^ ^]^

## 6 Clinical Evidence

Notwithstanding the promising results obtained from preclinical studies, the current evidence derived from randomized clinical trials suggests that regional anesthesia does not influence cancer progression.^[^ ^180^, ^181^, ^182^, ^183^, ^184^ ^]^ Similarly, a randomized clinical trial examining perioperative intravenous lidocaine infusion in patients undergoing pancreatic cancer surgery with curative intent failed to demonstrate any significant improvements in overall or disease‐free survival.^[^ ^143^ ^]^ Additionally, several researchers have examined the role of LAs when administered either locally or peritumorally within the context of oncological surgery, a method that may more accurately replicate conditions observed in preclinical studies, wherein LAs could potentially exert direct effects on tumor biology. (**Table** **2**).

**Table 2:** Clinical evidence of cancer outcomes with local anesthetic use.

Author [Year]	Cancer histology	Treatment group	Control group	Type	Sample size	Results [measure of association]
Melchi, et al. (1995)	Cutaneous melanoma	Local anesthesia	General anesthesia	Retrospective	LA, *n* = 150 GA, *n* = 215	No difference was observed in survival among groups after matching for prognostic variables (FRR 1.33; 95% CI 0.84–2.1)
Schlagenhauff, et al. (2000)	Cutaneous melanoma	Local anesthesia	General anesthesia	Retrospective	LA, *n* = 2185 GA, *n* = 2136	GA was associated with a decrease in 10‐year survival rate (RR 1.46; 95% CI 1.21–1.76; *p* < 0.0001).
Kofler, et al. (2018)	Lymph node dissection for cutaneous melanoma	Tumescent local anesthesia with 0.02% ropivacaine and 0.04% lidocaine	General anesthesia	Retrospective	LA, *n* = 119 GA, *n* = 162	No difference in 10‐year OS (56.2% vs 67.4%; p = 0.09) or DFS (43.8% vs 51.7%; *p* = 0.139).
MacFater, et al. (2020)	Colon adenocarcinoma	Intraperitoneal instillation with 75mg of ropivacaine followed by 3‐day intraperitoneal ropivacaine infusion (8 mg/hour)	Intraperitoneal instillation with normal saline	Secondary analysis of RCT	Ropivacaine, *n* = 18 Placebo, *n* = 19	No difference in all‐cause mortality (9 vs 7; *p* = 0.748). Ropivacaine intraperitoneal instillation was associated with cancer specific mortality (4 vs 0; p = 0.046)
Badwe, et al. (2024)	Breast cancer ^b)^	Peritumoral infiltration with 0.5% lidocaine	No placebo	RCT	Lidocaine, *n* = 786 No placebo, *n* = 797	Lidocaine peritumoral infiltration improved 5‐year DFS (HR 0.74; 95% CI 0.58–0.95; *p* = 0.017) and OS (HR 0.71; 95% CI 0.53–0.94; *p* = 0.019).

LA: Local anesthesia, GA: General anesthesia, FRR: Fatality rate ratio, CI: Confidence interval, RR: Relative risk, OS: Overall survival, DFS: Disease‐free survival, RCT: Randomized controlled trial, HR: Hazard ratio

^a)^ No information regarding local anesthetic studied

^b)^ No information regarding tumor histology

In an initial investigation comparing local to general anesthesia on cancer progression following melanoma surgery, Melchi et al. established that there was no association between the type of anesthesia used and oncological outcomes.^[^ ^185^ ^]^ Schlagenhauff et al. conducted a retrospective study using data from the Central Melanoma Registry of the Dermatological Society;^[^ ^186^ ^]^ their results indicated that general anesthesia for the primary excision of cutaneous melanoma was associated with lower survival (risk ratio 1.46; *p* < 0.0001; *n* = 4329) compared to local anesthesia. Additionally, in a retrospective study that involved 281 patients with cutaneous melanoma, Kofler et al. examined the association between tumescence local anesthesia and general anesthesia in relation to lymph node recurrence at the dissection site.^[^ ^187^ ^]^ Although the distant metastasis‐free survival rate was marginally lower in the tumescence local anesthesia group (64%) than in the general anesthesia group (49.9%, p = 0.025), no significant differences were observed in the melanoma‐specific survival, disease‐free survival, or lymph node‐free survival rates.

A randomized controlled trial by Badwe et al. reported that the administration of peritumoral 0.5% lidocaine, when given 7–10 min prior to incision, in comparison to no intervention, resulted in significantly increased disease‐free survival (hazard ratio [HR] = 0.74, 95% confidence interval [CI] = 0.58–0.95) and overall survival (HR = 0.71, 95% CI = 0.53–0.94) in women undergoing surgery for early breast cancer (*n* = 1583).^[^ ^188^ ^]^ The study found no significant differences in the rates of locoregional or distant recurrence. Although a limitation of Badwe's work was the absence of a placebo arm, the findings suggests that the local activity of anesthetics, rather than their systemic effects, exerts a positive influence in oncological outcomes.

MacFater et al. conducted a follow‐up analysis of a randomized controlled trial that compared the administration of intraperitoneal ropivacaine with normal saline in patients undergoing colonic resection for both benign and malignant diseases (*n* = 60).^[^ ^189^ ^]^ Among the patients, 37 had stage I‐III colon cancer, with 18 in the ropivacaine group and 19 in the placebo group. The study revealed no differences in locoregional recurrence and mortality rates and disease‐free survival, or overall survival rates. Nonetheless, cancer‐specific mortality was higher in the ropivacaine group than in the placebo group.

Several factors can explain the failure to translate preclinical findings into improved cancer outcomes. First, many in‐vitro and in‐vivo studies that show the anti‐tumor effects of local anesthetics utilize concentrations, dosages, or infusion rates that are significantly higher than those typically employed in clinical settings. For instance, Yang et al. reported increased survival in mice bearing bladder cancer when utilizing lidocaine concentrations of 500% (5 mg mL^−1^),^[^ ^190^ ^]^ while commonly used clinical concentrations range from 0.5% to 2%. Similarly, Xing et al. noted a reduction in hepatocarcinoma tumor growth in murine models with doses of 30 mg kg^−1^,^[^ ^191^ ^]^ in contrast to 5–7 mg kg^−1^ used for infiltration in clinical practice. Although Liu et al. administered clinically relevant doses of lidocaine alongside cisplatin intraperitoneally, their unusual dosing schedule (daily infusion for three days followed by a week off) further hinders the applicability of their results to clinical contexts.^[^ ^92^ ^]^ Second, preclinical models frequently do not accurately reflect human tumor behavior or the tumor microenvironment.^[^ ^192^ ^]^ Lastly, methodological shortcomings in preclinical studies, such as the absence of blinding, randomization, and standardized endpoints, may lead to biases that undermine their conclusions.^[^ ^193^ ^]^

In summary, the clinical evidence pertaining to the local or topical administration of LAs as an adjunct for cancer surgery is still inconclusive. Although certain studies indicate enhanced outcomes, these investigations are characterized by small sample sizes lacking sufficient blinding or are retrospective studies prone to confounding variables. While preclinical research consistently demonstrates that LAs have an effect on tumor biology, various factors hinder the effective translation of these findings into significant clinical advantages.

## 7 Conclusions

LAs show promising anticancer potential through various mechanisms, including antiproliferative, pro‐apoptotic, antiangiogenic, antimetastatic, anti‐inflammatory, and chemo‐sensitizing effects. These mechanisms operate through both VGSC‐dependent and ‐independent pathways. Additionally, LAs may influence cancer progression by modulating epigenetic regulation, post‐translational processes, miRNA expression, circular RNA expression, mitochondrial activity, and cellular metabolism. In vitro and in vivo studies support the role of LAs as potential adjuncts in cancer therapy. However, further large‐scale, randomized trials are needed to better understand the effectiveness of LAs when administered locally during cancer surgery and their impact on long‐term oncological outcomes. While we await the results of completed (NCT03594188, NCT02256228, NCT02012244, and NCT04510935) and ongoing clinical trials (NCT00938171, NCT01916317, NCT03813953, NCT02474511, and NCT04065009), it is advisable for patients undergoing oncological surgery to receive regional and local anesthesia to reduce pain, minimize the adverse effects of systemic analgesics, and improve recovery quality.

## Conflict of Interest

The authors declare no conflict of interest.
